# Fas and FasL gene polymorphisms are not associated with cervical cancer but differ among Black and Mixed-ancestry South Africans

**DOI:** 10.1186/1756-0500-2-238

**Published:** 2009-11-26

**Authors:** Koushik Chatterjee, Malin Engelmark, Ulf Gyllensten, Collet Dandara, Lize van der Merwe, Ushma Galal, Margaret Hoffman, Anna-Lise Williamson

**Affiliations:** 1 Division of Medical Virology and Institute of Infectious Disease and Molecular Medicine (IIDMM), University of Cape Town, Cape Town, Republic of South Africa; 2 Departments of Genetics and Pathology, Uppsala University, Uppsala, Sweden; 3 Division of Human Genetics, Faculty of Health Sciences, University of Cape Town, Cape Town, Republic of South Africa; 4 Biostatistics Unit, Medical Research Council, Cape Town, Republic of South Africa; 5 Department of Statistics, Faculty of Natural Sciences, University of Western Cape, Bellville, Republic of South Africa; 6 School of Public Health and Family Medicine, University of Cape Town, Cape Town, Republic of South Africa

## Abstract

**Background:**

Cervical cancer is one of the most important cancers in African women. Polymorphisms in the Fas (FasR) and Fas ligand (FasL) genes have been reported to be associated with cervical cancer in certain populations. This study investigated whether these polymorphisms are associated with cervical cancer or human papillomavirus (HPV) infection in South African women.

**Findings:**

Participants were 447 women with invasive cervical cancer (106 black African and 341 women of mixed-ancestry) and 424 healthy women controls, matched by age, (101 black African and 323 women of mixed-ancestry) and domicile (rural or urban). Two polymorphisms in Fas gene (FasR-1377G/A, FasR-670A/G) and one in FasL gene (FasL844T/C) were genotyped by TaqMan. None of the polymorphisms, or the Fas haplotypes, showed a significant association with cervical cancer. There was also no association with HPV infection in the control group. However, on analysis of the control group, highly significant allele, genotype and haplotype differences were found between the two ethnic groups. There were generally low frequencies of FasR-1377A alleles, FasR-670A alleles and FasL-844C alleles in black women compared to the women of mixed-ancestry.

**Conclusion:**

This is the first study on the role of Fas and FasL polymorphisms in cervical cancer in African populations. Our results suggest that these SNPs are not associated with cervical cancer in these populations. The allele frequencies of the three SNPs differed markedly between the indigenous African black and mixed-ancestry populations.

## Background

Cervical cancer is caused by persistent infection with specific types of human papillomavirus (HPV). During their life-time, many women become infected with HPV but most are able to clear the infection and only a small fraction develop cervical cancer. This indicates that additional risk factors play an important role in the development of cervical cancer. These risk factors include host and viral genetic factors as well as environmental and life style factors [1].

One of the most important mechanisms of the human immune system to control viral infection is to induce apoptosis of viral-infected cells [2]. Apoptosis is a highly regulated process of cell suicide and depends on signals from the cell surface death receptor Fas/CD95, which together with Fas ligand (FasL), triggers programmed cell death [3]. In this paper two polymorphisms in Fas (FasR-1377G/A, FasR-670A/G) and one in FasL (FasL844T/C) were studied in South African women with cervical cancer compared to controls without cancer of the cervix. The signals produced by the DNA binding of transcription factors SP1 (stimulatory protein 1) and STAT1 (signal transducer and activator of transcription 1) are associated with transcriptional activation and expression of the FasR gene. FasR-1377 and FasR-670 are located in SP1 and STAT1 binding sites, respectively. It has been shown that the FasR-1377A in the FasR promoter considerably reduces SP1 binding compared to -1377G, resulting in a decrease of Fas gene expression [4,5]. An A→G change at Fas-670 reduces the expression of the FasR, by abolishing the Gamma interferon activation signal binding site which results in decreased activation of induced cell death [4-6]. It has also been shown that the FasL-844C allele is associated with higher basal expression than the -844T allele and that reduced expression of FasL inhibits the apoptotic activity of the FasR-FasL pathway [7].

Six studies in different populations have been published on the association of these SNPs with cervical cancer. One investigated the FasR-670A/G polymorphism in a case-control study in the Chinese population and reported an association of the A allele and A/A genotype with higher risk of developing cervical cancer [8]. The same group showed an association of FasR-670A/A with cervical cancer in another case-control study [9] but did not find an association of the FasR-1377G/A or FasL-844T/C polymorphisms with disease severity. Two studies, one a case control study in a Polish group [10] the second an affected sib-pair (ASP) study in a Swedish group [11], did not detect an association with FasR-670A/G. Two case-control studies, one in the Chinese [12] and the other Swedish [13] populations, investigated the FasL-844T/C polymorphism. While the FasL-844C allele and C/C genotype were associated with cervical cancer in the Chinese study, no such effect was found in the Swedish women.

The importance of host genetic susceptibility factors for cervical cancer development has been demonstrated by epidemiological studies. A hereditary component of cervical tumors was detected in twin studies [14] and in a mother-daughter family study [15]. Biological first-degree relatives of women with cervical cancer have an increased risk of developing cancer of the cervix compared to non-biological relatives of women with cervical cancer [16]. Several studies addressing the association of cervical cancer with Fas and FasL have been published [8-13], but no studies have been performed in African populations. We therefore studied the association of polymorphisms in Fas and FasL with cancer of the cervix in South African women of black and mixed-ancestry origin.

## Methods

### Participants

Participants were 447 women with invasive cervical cancer (106 black African and 341 women of mixed-ancestry) and 424 controls (101 black African and 323 women of mixed-ancestry) without cancer of the cervix recruited from hospitals or clinics in the Western Cape Province, South Africa. The control women were selected to be representative of the population from which the cases were selected, specifically similar to the cases with respect to age, ethnicity and domicile status (urban/rural), so that case-control status would not be associated with either of those. There were 264 (59%) urban cases and 183 (41%) rural cases compared to 247 (58%) urban controls and 177 (42%) rural controls. The cases and controls formed part of a study to investigate the association of oral contraceptives with cervical cancer [17,18].

### Clinical specimens

Blood was collected from cases and controls, following written informed consent and stored at -80°C. The study was approved by the University of Cape Town Research Ethics Committee (REC REF: 075/2009). The subject identifiers were permanently unlinked from the specimens. The HIV infection status was known for 98% (n = 437) of the cases and 94% (n = 398) of the controls and 5% of the cases and 4% of the controls were positive for HIV infections. 13% of the controls were positive for HPV by Digene Hybrid Capture II. 14.5% of the controls in our study had abnormal cervical cytology.

### Extraction of genomic DNA

The genomic DNA was extracted using TotalNucleicAcid Extraction kit for MagNA Pure Compact nucleic acid extractor (Roche Diagnostics, Germany).

### Determination of FasR/FasL polymorphisms

Reactions were conducted in 5 μl volumes and contained 10 ng DNA, 1.75 μl PCR MasterMix (Applied Biosystems), 0.087 μl TaqMan assay mix (FasR-1377 (rs2234767, assay id, C_12123966_10), FasR-670 (rs1800682, assay id, C_9578811_10), FasL844 (rs763110, custom-designed)) (Applied Biosystems) and 1.16 μl distilled water. Cycling conditions on the ABI prism 7900 HT (Applied Biosystems) were 2 minutes at 50°C, 10 minutes at 95°C, followed by 40 cycles of 15 seconds at 92°C and one minute at 60°C. End point fluorescence was measured immediately after cycling. Alleles were assigned using SDS 2.0 software (Applied Biosystems) [19]. The genotyping was cross-checked by sequencing 4 samples for each polymorphism.

### Statistical Analysis

The genotype distributions were tested for Hardy-Weinberg equilibrium and linkage disequilibrium in cases and controls. Logistic regression was used to test for genotype, allelic and haplotype associations with cervix cancer status as well as baseline characteristics (age, ethnicity and smoking status) and secondary outcomes such as HIV status, abnormal cytology (Pap smear) and high risk HPV according to Hybrid Capture II HPV Test in the control group.

We inferred haplotypes from the two polymorphisms in FasR, and tested their association with cervix cancer status and between ethnicities in the controls.

Statistical analyses were done with R, the freely available environment for graphics and statistics http://www.r-project.org, and R packages dgc-genetics http://www-gene.cimr.cam.ac.uk/clayton/software/ and haplo.stats http://CRAN.R-project.org/package=haplo.stats. Broadly, haplo.stats uses the expectation maximisation (EM) algorithm, to calculate maximum likelihood estimates of probabilities, of haplotype pairs (one paternal, one maternal) for each subject, as described in Schaid et al. [20].

## Results

The mean age for black cases was 43.8 yrs (SD 9.2) and for mixed-ancestry cases it was 45.9 yrs (SD 8.1). The mean age for black controls was 42.9 yrs (SD 9.0) and for mixed-ancestry controls it was 45.6 yrs (SD 8.2). No significant differences in age or HIV status were observed between cases and controls.

The observed genotype frequencies of FasR-1377G/A, FasR-670A/G and FasL-844T/C in the controls were all in Hardy-Weinberg equilibrium (FasR-1377: P = 0.20, FasR-670: P = 0.58, FasL-844: P = 0.12). The two FasR polymorphisms were in tight linkage disequilibrium (LD) (P < 0.05), except in the black controls (P = 0.078).

Neither the allelic, nor the genotype frequencies showed a significant association (global P-values, 0.55, 0.12 and 0.77 for FasR-1377A, FasR-670A and FasL-844T respectively) with cervical cancer status (Table 1). The haplotype analysis for FasR-1377/-670 also did not reveal any association with cervical cancer in either the African black (global P = 0.1130) (Black) or the mixed-ancestry (global P = 0.7043) population (Table 2). The frequency of -1377A was 12% in black cases and 9% in the black controls, 21% in mixed-ancestry cases and 19% in mixed-ancestry controls. -670A was 18% in black cases and 24% in black controls, 36% in mixed-ancestry cases and 35% in mixed-ancestry controls. -844C was 17% in black cases and 14% in black controls, 34% in both mixed-ancestry cases and controls (Table 1).

**Table 1:** Counts (n), frequencies (%) and association statistics for genotypes and minor-alleles (FasR-1377G/A, FasR-670A/G, FasL-844T/C).

	Black		Mixed-ancestry		Genotype-cervical cancer association, adjusted for ethnicity		Genotype-ethnic association, in controls only	
	Cases	Controls	Cases	Controls				
Genotypes	n (%)	n (%)	n (%)	n (%)	P-value	OR (95% CI)	P-value	OR (95% CI)
**FASR-1377 G→A**								
GG	74 (77)	75 (83)	186 (64)	196 (66)	-	1	-	1
AG	21 (22)	14 (16)	87 (30)	86 (29)	0.428	1.14 (0.83-1.58)	0.007	0.43 (0.22-0.77)
AA	1 (1)	1 (1)	17 (6)	13 (4)	0.391	1.37 (0.67-2.87)	0.130	0.20 (0.01-1.04)
A allele	23 (12)	16 (9)	121 (21)	112 (19)	0.275	1.15 (0.89-1.49)	0.003	0.43 (0.24-0.72)
								
**FASR-670 A→G**								
GG	67 (65)	54 (55)	147 (44)	136 (42)	-	1	-	1
AG	35 (34)	43 (43)	132 (40)	146 (45)	0.099	0.79 (0.59-1.05)	0.207	0.74 (0.46-1.18)
AA	1 (1)	2 (2)	53 (16)	39 (12)	0.492	1.17 (0.74-1.87)	0.006	0.13 (0.02-0.44)
A allele	37 (18)	47 (24)	238 (36)	224 (35)	0.778	0.97 (0.79-1.19)	0.003	0.57 (0.39-0.82)
								
**FASL -844 T→C**								
TT	70 (68)	74 (74)	143 (44)	139 (44)	-	1	-	1
CT	31 (30)	23 (23)	144 (44)	136 (43)	0.515	1.1 (0.82-1.48)	< 0.001	0.32 (0.18-0.53)
CC	2 (2)	3 (3)	40 (12)	40 (13)	0.929	0.98 (0.61-1.57)	0.001	0.14 (0.03-0.41)
C allele	35 (17)	29 (14)	224 (34)	216 (34)	0.797	1.03 (0.83-1.27)	< 0.001	0.34 (0.22-0.51)

P-values and OR (95% confidence intervals) are for test of genotype or additive allelic association with cervix cancer risk, adjusted for ethnicity, and for genotype/additive association with ethnicity in controls. For genotypes, P-values next to genotype names are for joint model, others are for ORs of specific genotype compared to reference genotype, indicated with OR = 1. ORs for alleles are the odds for an additional copy of that allele. ORs for genotype-ethnicity association (in controls only) are odds of genotype/allele in black compared to odds of genotype/allele in individuals of mixed-ancestry.

Cases = Women with cancer of the cervix, Controls = Women without cancer of the cervix.

**Table 2:** Inferred frequencies, %, for FasR-1377 and FasR 670 haplotypes and odds ratios for risk of cervix cancer.

	Black			Mixed-ancestry		
		
Haplotype	Cases	Controls	OR (95% CI)	Cases	Controls	OR (95% CI)
GG	70	71	1	44	46	1
GA	18	20	0.8 (0.45-1.43)	36	35	1.08 (0.85-1.38)
AG	12	5	2.07 (0.87-4.93)	20	19	1.12 (0.83-1.52)
AA	0	4	-	0	0	-

Odds ratios (95% confidence intervals) are for testing FasR-1377 and FasR-670 haplotype association with risk of cervix cancer versus reference haplotype GG, stratified by ethnicity. Global P = 0.1130 (Black) and 0.7043 (Mixed ancestry).

The association of three SNPs with ethnicity was investigated only in the control group as the controls represent the larger South-African population. In the control group, all three SNPs showed significant allelic and genotype differences (global P-values, 0.004, 0.001, < 0.001 for FasR-1377A, FasR-670A and FasL-844T respectively) between ethnic groups (Table 1). The FasR haplotypes (FasR-1377/-670) also showed a significant difference (global P-value < 0.001) between the two ethnic groups among controls (Table 3). In the control group, both the GA and AG (FasR-1377 and FasR-670) haplotypes were more frequent in the mixed-ancestry group relative to African black group (AG: 19% compared to 5%; GA: 35% compared to 20%), while the GG haplotype was more common in blacks (71%) as compared to the mixed-ancestry group (46%).

**Table 3:** Inferred frequencies, %, for FasR-1377 and FasR-670 haplotypes and odds ratios for comparing ethnicity in the control group.

Haplotype	Black	Mixed-ancestry	OR (95% CI)
GG	71	46	1
GA	20	35	0.45 (0.31-0.67)
AG	5	19	0.33 (0.19-0.58)
AA	4	0	-

Odds ratios (95% confidence intervals) are odds of being black compared to mixed-ancestry, for FasR-1377 and FasR 670 haplotypes versus reference haplotype GG, in the control group. Global P-value < 0.001.

No significant association was found between any of the secondary outcomes (HIV infection, abnormal cytology (Pap smear) and hybrid capture high risk HPV) and any of the three SNPs in the control group, adjusted for ethnicity (results not shown here).

## Discussion

Polymorphisms in the Fas or FasL genes that impair the apoptotic signals have been associated with susceptibility to a number of different cancers [21-27] including cervical cancer [8-13]. We are the first to study the frequency of the FasR-1377G/A, FasR-670A/G and FasL844T/C polymorphisms in an indigenous black African as well as in a mixed-ancestry population and found no association with cancer of the cervix. The tendencies of the odds ratios (ORs) (FasR-1377A/A 1.37, FasR-670A/A 1.17) point towards an increasing susceptible effect and a need for a larger study. Although the result was not statistically significant, there were more cases with FasR-1377A variant compared to controls in both black African (12% vs. 9%) and mixed-ancestry (21% vs. 19%) women. Our results agree with the lack of association of Fas SNPs with cervical cancer shown by three other reports [10,11,13]. The differing results between studies might be due to one or several factors, including the difference in ethnic origin of populations studied and the difference in sample size. The presence of another causative mutation in tight LD with any of these three polymorphisms can also not be ruled out.

We observed statistically significant allele frequency differences for all the three Fas SNPs between ethnic groups in the control women. For FasR-1377G/A, the A allele frequency was 9% in the black Africans and 19% in women of mixed-ancestry, which were both markedly lower than the frequency in other populations, such as the 43% and 31% in Taiwanese and Chinese populations, respectively [8,12]. The FasR-670A allele frequency was 24% in black Africans and 35% in women of mixed-ancestry. This is again lower than the average of 53% for populations from Europe, Australasia and Asia [4,8,10,28,29]. The frequency of the FasL-844C alleles was 14% in black women and 34% in women of mixed-ancestry, which are very low compared to the 69% and 71% in the Chinese and Swedish populations respectively [12,13]. The unusually low frequency of the Fas and FasL alleles in the African population could contribute to a lower power to detect an association in the present study.

The FasR-1377G/A and FasR-670A/G SNPs are in tight LD in our population. Both the AG and GA haplotypes were significantly more frequent in women of mixed-ancestry compared to GG, the most common haplotype, which was observed at a higher frequency in black women (Table 3).

## Conclusion

Our study did not show a significant association of the FasR-1377G/A, FasR-670A/G and FasL-844T/C polymorphisms with cervical cancer. A statistically significant difference was found in the allele and genotypes frequencies of the three SNPs between women of black African and mixed-ancestry in the control population.

## Competing interests

The authors declare that they have no competing interests.

## Authors' contributions

KC participated in planning of the study, performed extraction of DNA, genotyping, analyzing the results and basic statistical analysis. ME helped in genotyping, analysis and interpretation of the results. CD helped in reporting and interpreting of the results. LM and UGA performed advanced statistical analysis and helped with reporting and interpretation of results. MH coordinated the collection of the biological samples. UGY helped in planning the study and supervised the genotyping part. ALW was responsible for the storage of the biological samples, helped in planning and supervising the study and helped in interpretation of the results. All authors critically read and took part in finalizing the manuscript.
